# Variations in ventricular distribution of polymyxin B following intraventricular administration in patients with central nervous system infections

**DOI:** 10.1128/aac.00943-25

**Published:** 2025-11-06

**Authors:** Huifang Jiang, Yangmin Hu, Jing Cai, Jiali Zhang, Meng Chen, Lvfeng Hu, Xuben Yu, Kai Wei, Haibin Dai, Wei Yan

**Affiliations:** 1 Department of Pharmacy, The Second Affiliated Hospital, Zhejiang University School of Medicine 89681 , Hangzhou, China; 2 Department of Neuroscience Care Unit, The Second Affiliated Hospital, Zhejiang University School of Medicine 89681 , Hangzhou, China; 3 Department of Pharmacy, The First Affiliated Hospital of Wenzhou Medical University 89657, Wenzhou, China; 4 Department of Imaging, The Second Affiliated Hospital, Zhejiang University School of Medicine 89681 , Hangzhou, China; 5 Department of Neurosurgery, The Second Affiliated Hospital, Zhejiang University School of Medicine 89681 , Hangzhou, China; Houston Methodist Hospital and Weill Cornell Medical College, Houston, Texas, USA

**Keywords:** polymyxin B, central nervous system infection, intraventricular, gram-negative bacteria, carbapenem-resistant gram-negative bacteria

## Abstract

Polymyxin B (PMB) demonstrates activity against multidrug-resistant Gram-negative pathogens. Intraventricular administration is considered when systemic therapy yields suboptimal cerebrospinal fluid (CSF) levels. We describe two cases of carbapenem-resistant gram-negative meningoencephalitis successfully treated with intraventricular PMB, in which unilateral PMB administration appeared to achieve bilateral ventricular distribution, with higher concentrations on the administration side. A potential association was observed between reduced CSF drainage and increased PMB concentrations. These findings may offer preliminary insights into optimizing PMB dosing strategies for CNS infections.

## INTRODUCTION

Central nervous system (CNS) infections caused by Gram-negative bacilli (GNB) can lead to severe, often life-threatening conditions, with mortality rates reaching as high as 71% (1). The emergence of carbapenem-resistant GNB (CRGNB) strains has further complicated the treatment of CNS infections. According to the 2022 CHINET report (http://www.chinets.com), *Acinetobacter baumannii* and *Klebsiella pneumoniae* were detected in cerebrospinal fluid (CSF) cultures at rates of 11.5% and 11%, respectively. Notably, these strains exhibit varying degrees of resistance to carbapenems. Polymyxins, including colistin and polymyxin B (PMB), have emerged as last-line therapies for CRGNB-related CNS infections and have demonstrated efficacy. However, the intravenous administration of polymyxins is constrained by their poor penetration into the CSF, with only approximately 5%–11% of the simultaneous plasma concentration reaching the CSF (2–4), thereby decreasing treatment efficacy and potentially promoting the development of antibiotic resistance. Because resistance to polymyxins during monotherapy is relatively common, they are often employed in combination with other antimicrobial agents when treating multidrug-resistant organisms (5, 6).

To overcome this challenge, intraventricular (IVT) or intrathecal (ITH) administration of polymyxins has been widely adopted. These routes have the benefit of enhanced CSF drug concentrations, effectively treating CRGNB infections while reducing the nephrotoxicity associated with intravenous administration (2). However, we acknowledge that concerns regarding the limited antibacterial activity of this intervention and the potential emergence of drug resistance associated with it remain unresolved. Given these outstanding concerns, further well-designed clinical evaluations are therefore warranted to comprehensively assess its actual efficacy and long-term safety profiles. In patients with CNS infections, factors such as inflammation, altered CSF secretion, hydrocephalus, and intracranial pressure changes can significantly impact drug distribution within the ventricles. A study conducted in mice employed mass spectrometry imaging and revealed that PMB predominantly accumulates in the lateral ventricle on the injection side while remaining undetectable in the contralateral lateral ventricle and surrounding brain tissue (7). This finding indicates a localized and specific distribution pattern. The distribution of PMB following ventricular administration for CNS infections in humans remains insufficiently understood. This report presents two cases of meningitis that showed different ventricular distribution of PMB following IVT injection of PMB.

The study protocol was approved by the Ethics Committee of the Second Affiliated Hospital of Zhejiang University School of Medicine (No. 2024-0181). Prior to sample collection, informed consent was obtained from the two patients. For critically ill or incapacitated inpatients unable to provide consent due to severe conditions, consent was obtained from their legal guardians.

## CASE PRESENTATION

### Patient 1

**Fig 1:**
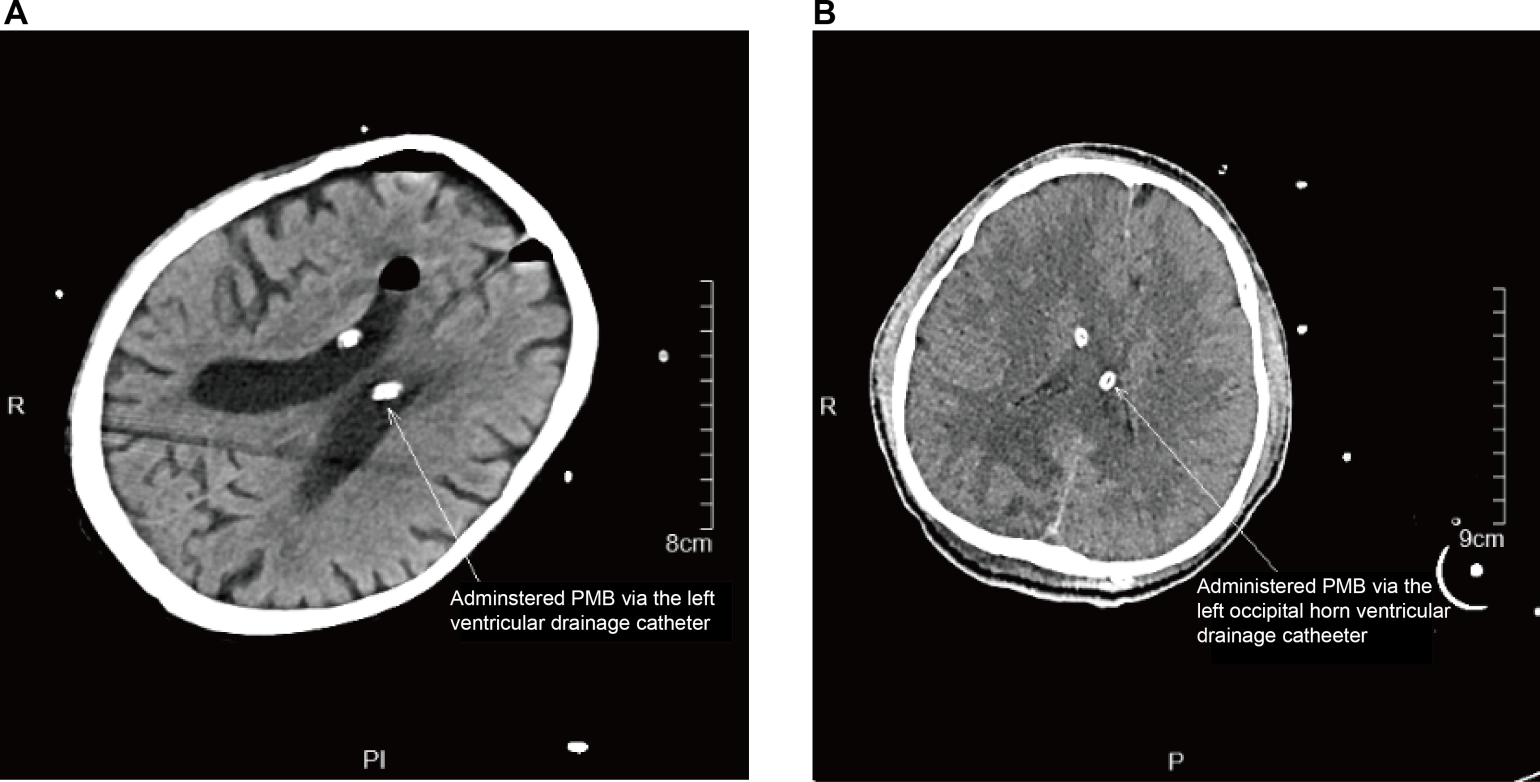
Head CT images of patients. (**A**) Head CT images of patient 1. The patient had EVDs placed in both lateral ventricles. PMB was administered through the left EVD from day 1 to day 3. On days 4 and 6, CSF samples were simultaneously collected through the bilateral ventricular drainage catheters to measure PMB concentrations in the CSF. (**B**) Head CT images of patient 2. Ventricular drainage catheters were placed in both the left occipital horn and the right frontal horn. PMB was administered via lumbar puncture on days 1 and 2, and through the left occipital horn drainage catheter on days 3 and 4. On day 5, after IVT administration, the drainage catheters were clamped for 1 hour, and CSF samples were collected 2 hours later from both the left occipital horn and right frontal horn drainage catheters.

A 68-year-old male was transferred to the Second Affiliated Hospital of Zhejiang University School of Medicine due to intracranial infection and was diagnosed with purulent meningoventriculitis. Due to the presence of multiple abscesses in both lateral ventricles, bilateral external ventricular drainage (EVD) was performed to ensure adequate CSF drainage and facilitate targeted infection control. The drainage tubes were placed in the left and right lateral ventricles (Fig. 1A). The CSF culture identified carbapenem-resistant *K. pneumoniae* (CRKP), which was sensitive to PMB with a minimum inhibitory concentration (MIC) of 0.5 µg/mL. Pre-treatment CSF analysis showed glucose at ≈ 20.7 mg/dL (1.15 mmol/L), total protein 445.9 mg/dL (≈ 4.46 g/L), nucleated cell count 159 cells/µL (159 × 10⁶/L) with 92% neutrophils, and opening pressure >330 mmH₂O (≈ 33.0 cmH₂O; ≈ 24.3 mmHg).

All treatment regimens were adjusted by the attending physicians based on the patient’s condition. IVT administration of PMB was initiated at a daily dose of 50,000 units, delivered through the left ventricular drainage catheter every day. The administration was performed at the same time each day, with a fixed interval of 24 hours between doses. Before each PMB administration, 5 mL of CSF was aspirated and discarded to prevent an increase in intracranial pressure caused by the PMB bolus. The PMB was injected, and CSF samples were collected under sterile conditions using a needleless connector in the ventricular drainage system, which was disinfected with iodine prior to use. Following administration, the ventricular system was flushed immediately with 2 mL of saline solution to minimize residual PMB in the drainage system. Both EVD systems were kept closed for at least 1 hour, provided intracranial pressure remained below 272.16 mmH₂O (≈20 mm Hg). If intracranial pressure exceeded this threshold, the drainage was opened.

The PMB concentrations in CSF were evaluated after at least 3 days of treatment. On the fourth and sixth days, CSF samples were simultaneously collected through the bilateral ventricular drainage catheters at fixed time points prior to IVT administration, to measure PMB concentrations in the CSF. The quantification of PMB concentration in the CSF was carried out using a validated high-performance liquid chromatography-tandem mass spectrometry (HPLC-MS/MS) method, as described by Wang and Liu et al. (1) (1, 8). The calibration range for PMB was established at 0.01–20 µg/mL. The validation of this method, which included assessments of the calibration curve, selectivity, accuracy, precision, matrix effect, recovery, and stability, complied with FDA standards. On day 4, the concentration of PMB in the left lateral ventricle was 4.12 µg/mL, while it was 1.48 µg/mL in the right lateral ventricle; both values exceeded the MIC. The ventricular drainage volume on that day was 110 mL (60 mL on the left side and 50 mL on the right side). On day 6, PMB concentrations were 2.52 µg/mL on the left side and 1.61 µg/mL on the right side. The ventricular drainage volume on that day was 135 mL (93 mL on the left and 42 mL on the right).

**Fig 2:**
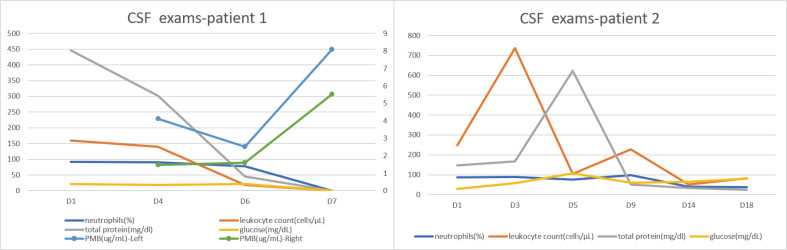
Serial CSF monitoring of cells, proteins, glucose, and PMB concentrations (left/right ventricles) since treatment start in patient 1 and patient 2.

Following the removal of the right drainage tube on the afternoon of day 6, the PMB trough concentration in CSF collected from the left side on day 7 (D7) was 8.09 µg/mL. Four hours after administration, the PMB concentration in the CSF was measured at 5.52 µg/mL. The total CSF drainage from the left side on D7 was 63 mL. All these measurements and their detailed recording are presented in (Table 1). CSF cultures obtained on days 4, 6, and 7 were negative, indicating initial microbial control. The corresponding dynamic changes in CSF biochemical parameters (white cell count, total protein, and glucose) are depicted in Fig. 2. Subsequently, the patient achieved complete microbial clearance in the CSF, which was accompanied by significant improvement in intracranial infection symptoms. The clinical stabilization allowed for the patient’s transfer to a rehabilitation hospital for continued treatment and recovery.

**TABLE 1:** PMB concentration in the CSF and clinical data of two patients with meningitis^*a*^

Patient 1									
Pathogen	MIC for PMB	Location of administration	Day of PMB treatment	Sampling location	Time after IVT admission (h)	PMB(μg/mL)	CSF drainage/day (mL)	Microbiology	ICU outcome
CRKP	0.5	LLV drainage	D4	LLV drainage	0	4.12	60	Cure	Survival
				RLV drainage	0	1.48	50		
			D6	LLV drainage	0	2.52	93		
				RLV drainage	0	1.61	42		
			D7	LLV drainage	0	8.09	63		
				LLV drainage	4	5.52	/^*b*^		
Patient 2									
CRAB	2	LLV drainage	D5	LOH drainage	2	267.64	/	Cure	Survival
				RFH drainage	2	10.38	/		

^*a*^ PMB, polymyxin B; MIC, minimum inhibitory concentration; IVT, intraventricular; CSF, cerebrospinal fluid; ICU, intensive care unit; LLV, left lateral ventricle; RLV, right lateral ventricle; LOH, left occipital horn of the lateral ventricle; RFH, right frontal horn of the lateral ventricle; CRKP, carbapenem-resistant *Klebsiella pneumoniae*; CRAB, Carbapenem-resistant *Acinetobacter baumannii*.

^*b*^ The forward slash (/) indicates that no data are available for that specific entry.

These findings suggest that unilateral intraventricular administration of drugs may allow distribution to the contralateral ventricle. Nevertheless, drug concentrations tended to be higher on the administration side than on the opposite side. Furthermore, a trend toward higher drug concentrations was observed with lower CSF drainage volumes. Notably, the removal of the contralateral drainage catheter appeared to influence drug distribution, underscoring the complex and dynamic factors that may affect intraventricular drug delivery in CNS infections. Although seizures and chemical ventriculitis have been reported as potential adverse effects following intraventricular administration of PMB (9). Importantly, no adverse reactions were observed in this patient during the course of intraventricular therapy. At the most recent follow-up—1 year after intrathecal administration—the patient had recovered well, with no evidence of seizures, chemical ventriculitis, or other adverse effects and remains under outpatient monitoring.

### Patient 2

A 21-year-old male presented to a local hospital after a fall from a height, resulting in impaired consciousness for approximately 9 hours. The patient underwent endotracheal intubation and was diagnosed with severe traumatic brain injury following a comprehensive evaluation. For further treatment, the patient was transferred to the Second Affiliated Hospital of Zhejiang University School of Medicine. Upon admission, the patient underwent intracranial hematoma evacuation, decompressive craniectomy, and EVD. On postoperative day 8, the patient developed a fever, with a maximum temperature of 39.6°C, and a significant deposition of white flocculent material was observed in the drainage tubes. CSF analysis revealed a leukocyte count of 7,160 cells/µL (7,160 × 10⁶/L), 90% neutrophils, glucose 52.1 mg/dL (2.89 mmol/L), and protein >300 mg/dl (>3.0 g/L). Due to obstruction of CSF drainage and intracranial infection, the patient underwent a second EVD procedure involving the left occipital horn and bilateral frontal horns (Fig. 1B). This was performed to improve CSF circulation after obstruction; the newly placed bilateral drainage catheters were confirmed to be patent and functioning. Nonetheless, fever persisted postoperatively, reaching a maximum temperature of 38.3°C. Repeat CSF analysis revealed a leukocyte count of 247 cells/µL (247 × 10⁶/L) with 87% neutrophils, glucose at ≈ 29.7 mg/dL (1.65 mmol/L), and total protein 147.9 mg/dL (≈ 1.48 g/L). CSF culture identified carbapenem-resistant *A. baumannii* (CRAB) with a PMB MIC of 2 µg/mL. On days 1 and 2, PMB was administered via lumbar puncture at a dose of 50,000 units. On days 3 and 4, PMB (50,000 units) was administered through the left occipital horn drainage catheter. On day 5, after IVT administration, the drainage catheters were clamped for 1 hour, and CSF samples were collected 2 hours later from both the left occipital horn and right frontal horn drainage catheters. The PMB concentration in the CSF was measured at 267.64 µg/mL on the left and 10.38 µg/mL on the right. The detailed data records of these measurements can be found in . CSF cultures on days 3, 5, 9, 14, and 18 were negative. The corresponding CSF biochemical parameters (white cell count, total protein, and glucose) are shown in Fig. 2. Subsequently, the patient achieved microbial clearance in the CSF, with symptom improvement and a stabilized condition. The patient was then transferred to a rehabilitation hospital for further recovery and treatment.

These findings indicate that 2 hours after intraventricular administration, both the left occipital horn and right frontal horn drainage catheters had detectable and relatively high drug concentrations. Still, the concentration was significantly higher on the left side compared to the right, which may be related to the positioning of the ventricular drainage catheters. Notably, throughout the treatment period and follow-up, no adverse reactions, including seizures or chemical ventriculitis, were detected. At the 1-year follow-up, the patient maintained clinical stability and continues to be under regular outpatient monitoring.

All treatment regimens were adjusted by the attending physicians based on the patients’ conditions. For both cases, IVT/ITH administration of PMB was performed using the preparation from Shanghai First Biochemical Pharmaceutical Co., Ltd. (each vial containing 50 mg of PMB, equivalent to approximately 500,000 units; 1 mg ≈ 10,000 units), at an initial daily dose of 50,000 units.

## DISCUSSION

Our cases add three practical and novel contributions to the current literature. First, we preliminarily investigate the intraventricular distribution of PMB after intraventricular or intrathecal administration, offering insights into its spatial disposition within the ventricular system. Second, although many previous case series (9–13) have reported clinical outcomes and dosing regimens, they rarely offered paired pharmacokinetic or therapeutic drug-monitoring (TDM) data. In contrast, we provide time-matched CSF MICs together with repeated CSF biochemical measurements. Third, recent studies (1) have underscored substantial inter- and intra-individual variability in PMB concentrations in the CSF and have called for more TDM-guided information. Our findings demonstrate this variability within a single patient and correlate it with drug levels and drainage status, thereby supplying a reference for clinical dose adjustments.

While it is commonly believed that IVT administration alone results in uniform drug distribution throughout the ventricles, this hypothesis has not been adequately studied in clinical settings. The measurement of drug concentrations in the CSF provides a valuable reference for evaluating this distribution. These cases provide valuable insights into the factors influencing drug distribution in the CSF following IVT administration. In both cases, unilateral IVT administration resulted in the drug reaching the contralateral ventricle. However, previous animal studies have reported conflicting results (7). Notably, both cases were part of a prospective clinical study investigating the use of PMB via intraventricular administration alone; however, given the very limited sample size and absence of a control group, the observations should be interpreted as hypothesis-generating rather than confirmatory. The patients received IVT PMB without concurrent intravenous administration. Both patients achieved clinical cure and were successfully discharged. During subsequent follow-up, no adverse reactions were observed, further supporting the safety and effectiveness of IVT monotherapy in these instances. Moreover, drug concentrations tended to be relatively higher on the administered side compared to the opposite side, possibly indicating uneven distribution of the drug in the CSF following IVT administration. This unevenness may reflect loculated CSF compartments or impaired communication between ventricles due to inflammation, even when imaging suggests otherwise, a phenomenon that merits further validation in larger prospective PK/PD studies. These results appear to align with previous studies, which have reported higher and more uniform concentrations in both the lumbar and ventricular CSF after IVT administration of aminoglycosides. In contrast, ITH administration has been associated with more variable drug distribution, often resulting in significantly lower and potentially inadequate concentrations in the ventricular CSF (14). Additionally, our cases suggest that the concentration of the drug in the CSF may be considerably affected by the volume of drainage. Reduced CSF drainage leads to increased drug retention and ventricular concentration. Additionally, removal of the drainage catheter may further alter drug distribution and concentrations, highlighting the critical role of drainage management in intraventricular therapy. These findings are consistent with previous reports stating that reduced drainage improves drug retention and enhances intraventricular drug concentrations (15). Although these associations are biologically plausible, the current evidence is insufficient to establish causality, and future controlled studies are required to quantify the impact of drainage volume and catheter management on intraventricular drug exposure.

Furthermore, our cases seem to indicate that catheter positioning may have a notable impact on drug distribution. It appears that drugs tend to accumulate in the lower segments of the ventricles, suggesting the importance of appropriate catheter placement to potentially ensure effective and uniform drug delivery.

In the management of CNS infections, which are serious complications commonly associated with neurosurgical procedures, these findings highlight the effectiveness of IVT admission of PMB. This approach achieves drug concentrations that exceed the MICs of susceptible bacteria, facilitating rapid sterilization of the CSF in cases where systemic antibiotics alone are insufficient (2). Although IVT or ITH administration of polymyxins has been increasingly applied in clinical practice, the use of IVT/ITH therapy alone remains uncommon. Most studies suggest that local administration is best utilized as an adjunct to intravenous therapy, particularly for multidrug-resistant GNB (MDR-GNB) infections, where combination therapy achieves higher CSF sterilization rates and improved outcomes (16, 17). However, the two cases in our study—where patients achieved both microbiologic and clinical cure with intraventricular PMB alone (without systemic therapy)—add to the existing evidence from prior case series and reports (9–13): these findings collectively indicate that IVT/ITH monotherapy may be a viable option in select clinical scenarios (e.g., isolated ventriculitis or uncomplicated meningitis, as noted in relevant literature), where direct local delivery can achieve sufficient CSF drug levels. Given the limited data on this approach, careful patient selection, close clinical and microbiologic monitoring, and further validation in larger studies remain essential to define its role in routine practice.

PMB is a concentration-dependent antibiotic, with the area under the concentration-time curve to MIC ratio (AUC/MIC) being the most reliable predictor of its efficacy. Current recommendations suggest that an AUC_0–24_ of approximately 50 h·μg/mL at steady state is required for optimal therapeutic effect. This corresponds to a target average steady-state plasma concentration (Css, avg) of approximately 2 μg/mL (18). Our cases support that after IVT administration, CSF drug concentrations consistently exceeded 2 µg/mL, surpassing the breakpoint for GNB, indicating the effectiveness of this administration method.

The guidelines recommend a daily IVT dose of 5 mg PMB for the treatment of Gram-negative ventriculitis and meningitis (16). However, its distribution is influenced by several factors, including ventricular size, CSF drainage volume, drug distribution volume, and CSF clearance capacity, resulting in significant interindividual variability (18). Following IVT or ITH administration of PMB, the guidelines suggest that the target drug concentration in the ventricles should be 10–20 times the MIC of the causative pathogen (16). However, further research with larger sample sizes is required to confirm these recommendations. Additionally, some studies advise adjusting the IVT/ITH PMB dosage based on the daily CSF drainage volume. For a drainage volume of 50–200 mL/24 hours, a daily dose of 50,000 units is recommended (19). In the two cases presented, the daily CSF drainage volumes fall within this range, highlighting the promising potential and feasibility of this dosing strategy. Nevertheless, in clinical practice, fluctuations in CSF drainage volumes were observed. In cases with a drainage volume that is less than 50 mL or greater than 200 mL, the optimal dosage remains unclear and warrants further investigation.

In conclusion, unilateral intraventricular administration of PMB successfully delivers the drug to the contralateral ventricle, with consistently higher concentrations detected on the side of administration. The concentration of the drug in the CSF appears to be influenced by the CSF drainage volume, with the drug tending to accumulate in lower-positioned areas. Given the exploratory nature and small scale of this study, these findings should be regarded as hypothesis generating. They provide a foundation for future prospective, controlled studies aimed at elucidating the pharmacokinetic determinants of intraventricular PMB therapy and optimizing dosing strategies for different drainage conditions and catheter configurations. Further prospective studies are needed to better understand the dosage-related factors associated with the intraventricular administration of PMB.

## CHALLENGE QUESTION

Which of the following therapeutic considerations is most important when administering intraventricular PMB for the treatment of CNS infections caused by multidrug-resistant GNB?

1. Systemic administration alone is sufficient to achieve therapeutic CSF levels of PMB in most cases.
2. PMB is distributed evenly throughout the ventricular system regardless of the administration site.
3. Reduced CSF drainage via EVD may increase CSF PMB concentration.
4. PMB cannot cross to the contralateral ventricle when administered unilaterally.
5. Increasing CSF drainage improves bacterial clearance by reducing drug concentration variability.
